# A 30-m annual corn residue coverage dataset from 2013 to 2021 in Northeast China

**DOI:** 10.1038/s41597-024-02998-7

**Published:** 2024-02-16

**Authors:** Yi Dong, Fu Xuan, Xianda Huang, Ziqian Li, Wei Su, Jianxi Huang, Xuecao Li, Wancheng Tao, Hui Liu, Jiezhi Chen

**Affiliations:** 1 College of Land Science and Technology, China Agricultural University, Beijing, 100083 China; 2 Key Laboratory of Remote Sensing for Agri-Hazards, Ministry of Agriculture and Rural Affairs, Beijing, 100083 China

**Keywords:** Carbon cycle, Sustainability

## Abstract

Crop residue cover plays a key role in the protection of black soil by covering the soil in the non-growing season against wind erosion and chopping for returning to the soil to increase organic matter in the future. Although there are some studies that have mapped the crop residue coverage by remote sensing technique, the results are mainly on a small scale, limiting the generalizability of the results. In this study, we present a novel corn residue coverage (CRC) dataset for Northeast China spanning the years 2013–2021. The aim of our dataset is to provide a basis to describe and monitor CRC for black soil protection. The accuracy of our estimation results was validated against previous studies and measured data, demonstrating high accuracy with a coefficient of determination (R^2^) of 0.7304 and root mean square error (RMSE) of 0.1247 between estimated and measured CRC in field campaigns. In addition, it is the first of its kind to offer the longest time series, enhancing its significance in long-term monitoring and analysis.

## Background & Summary

Crop residue cover is a vital measure for soil protection within agricultural sustainable development, especially for black soil protection in Northeast China which is an important grain-producing area with more than 30% of China’s corn yield^1,2^. Implementing crop residue covering after harvesting mitigates wind and water erosion, enhances soil organic carbon content and microbial populations, improves soil water retention capacity, and enhances physicochemical properties^3–5^. Global observations show that conservation tillage is the most eco-friendly tillage practice, can significantly reduce greenhouse gas(GHG) emissions, improve crop yields under certain circumstances, and increase soil microbial diversity and soil organic carbon^6^. Crop residue cover, as an alternative to the conventional method of straw disposal, offers an effective approach to mitigate air pollution and minimize harmful emissions. Conservation tillage is defined as the crop residue coverage of more than 0.3 by the Food and Agriculture Organization of the United Nations (FAO)^7^. Estimating crop residue coverage is essential for the conservation tillage system, which is one important input for many agricultural ecological models^8^. Therefore, it is vital to estimate crop residue coverage on a large scale quickly and accurately.

The traditional ways of estimating crop residue coverage include visual judgment or measuring using a band tape in a field campaign. However, these direct measurement or photography approaches are laborious, time-consuming, and not conducive to large-scale implementation due to their inherent discontinuity^9,10^. Remote sensing technique has become a popular method for crop residue coverage estimation because of its high spatial coverage and temporal revisit on a large scale^11,12^. The most widely used method for crop residue coverage estimation by remote sensing is developing the correlation model between measured data in field campaigns and spectral residue indices using parametric and nonparametric methods. The commonly used spectral indices include Normalized Difference Senescent Vegetation Index (NDSVI)^13^, Normalized Difference Residue Index (NDRI)^14^, Normalized Difference Tillage Index (NDTI)^15^, Shortwave Green Normalized Difference Index (SGNDI)^16^, Shortwave Infrared Normalized Difference Residue Index (SINDRI)^17^, Broadband spectral Angle Index (BAI)^18^, Dead Fuel Index (DFI)^19^, Normalized Difference Index (NDI)^20^, Modified crop residue cover (MCRC)^16^, Simple Tillage Index (STI)^15^ and Short-wave near-infrared Normalized Difference residue Index (SRNDI)^21^, etc. Since every spectral index has its advantages and disadvantages^22^, the combination of several indexes is popular for improving crop residue coverage estimation. Furthermore, the covering crop residues have varied and regular textural characteristics for they are managed by harvester. Therefore, the combination of spectral residue index and texture features will be used in this study, which has been proven the improve crop residue coverage^23^.

For the modelling of correlation between measured coverage and spectral indices and textual features, machine learning methods including random forests (RF), support vector machines (SVM) and artificial neural networks (ANN) have been popular especially. Ding *et al*.^24^ found that the estimation accuracy using machine learning methods was better than that using univariate regression, and the estimation accuracy of combining texture features with spectral information was higher than that of using spectral information alone. Zhu *et al*.^25^ and Dong *et al*.^26^ also prove the significance of machine learning methods with stable capability. Therefore, the Random Forest regression model is used for crop residue coverage estimation in this study.

Corn is the main crop in Northeast China, especially in Songnen Plain where is the Golden Corn Belt in China. Therefore, the CRC is estimated in this study based on our previous crop classification results^27^. This study proposes an approach to map the 30-m annual CRC dataset from 2013 to 2021 in Northeast China, and there are two maps after harvesting and before sowing in the next growing season for each year. To the best of our knowledge, this is the first CRC product in Northeast China, which is vital for monitoring conservation tillage to protect black soil in China. Specifically, by synthesizing Landsat archives and MODIS reflectance using HISTARFM to produce continuous reflectance images, we develop a sampling generation method involving measuring in field campaign, collecting from high resolution Google Earth images and unmanned aerial vehicle (UAV) images. Furthermore, the combination of spectral indices and textural features is optimized for random forest modelling to produce CRC in Northeast China from 2013 to 2021. Finally, the accuracy of 30-m annual CRC dataset from 2013 to 2021 in Northeast China is assessed using independent samplings. Meanwhile, we also compare our 30-m CRC dataset with the published dataset in Songnen Plain by Li *et al*.^28^. Furthermore, the official statistical data on the conservation tillage area of *China Agricultural Machinery Industry Yearbook and China Agricultural* Mechani*zation Yearbook* (https://data.cnki.net/yearBook/single?id=N2023060184) is used to validate this CRC dataset.

## Methods

### Study area

**Fig. 1:**
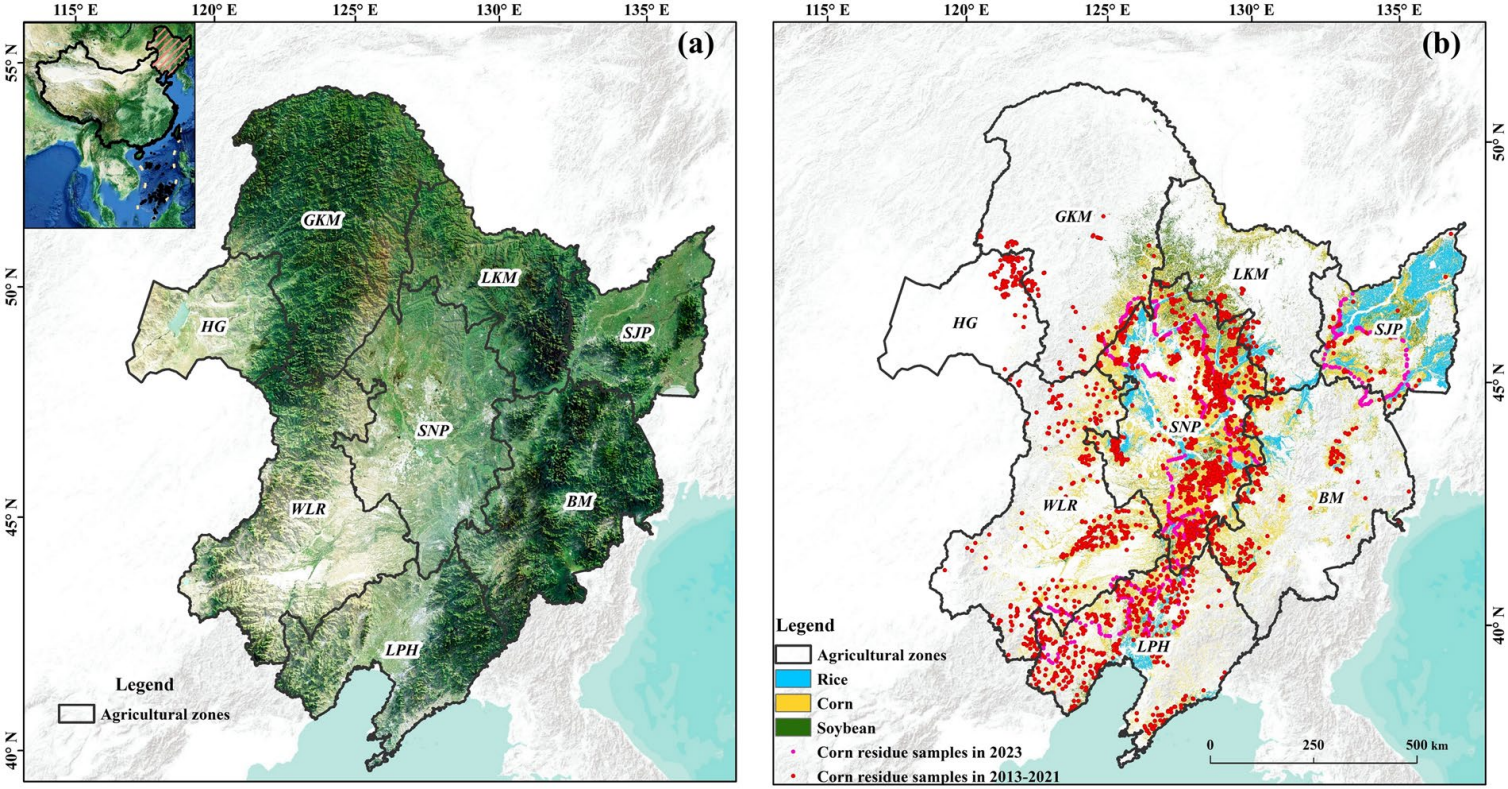
The study area of Northeast China (**a**) and samplings for CRC estimation (**b**).

The study area is located in Northeast China, spanning Heilongjiang, Jilin, Liaoning, and eastern Inner Mongolia, from latitude 38°26′ to 55°24 N and longitude 115°30′ to 135°8 E (Fig. 1). And the study area covers cold and moderate continental monsoon climate zones with an average annual temperature of −3.8–11.3 °C and precipitation varying between 298 and 880 mm. Considering the climate and precipitation amount, the study area can be divided into eight agricultural zones, including Sanjiang Plain Zone (SJP), Greater Khingan Mountain Zone (GKM), Lesser Khingan Mountain Zone (LKM), Baekdu Mountain Zone (BM), Songnen PlainZone (SNP), Liaoning Plain and Hilly Zone (LPH), Western Liao River Zone (WLR), and Hulunbuir Grassland Zone (HG). The Northeast China is with four distinct seasons, rain and heat over the same period, fertile black soil, where is an important region for grain production in China. The region is also suitable for monoculture cultivation from May to September each year with planted rice, corn, and soybeans mainly, and the corn planted area and production account for more than 30% of the total corn production in China^29^.

### Samples collection

#### Measuring in field campaigns

There are three extensive field campaigns used for CRC estimation in this study done on November 2020 (after harvesting), April 2021 (before sowing in the next growing season) and April 2023 (before sowing in the next growing season), respectively. Firstly, the sample plots with uniform straw cover were selected, within each quadrat (30 m × 30 m), there are 5 photos taken randomly and the mean of them is used for estimating CRC. The aim was to minimize the effect of shadows and ensure sample homogeneity. To achieve this, all photos were captured between 8:30 a.m. to 4:30 p.m., with the camera held at a height of 1.5 meters above the ground and in the opposite direction of the sun. All samples are located using a Huace i80 real-time kinematic (RTK) GPS receiver (Huace Ltd., Shanghai, China). For each photo, the CRC value is calculated using image segmentation. Meanwhile, the average value of five random photos in each quadrat is calculated as the CRC in each quadrat.

#### Calculating from UAV images

The high spatial resolution UAV images were collected during six field campaigns in 2015, 2019 and 2021, respectively. Two popular UAV models, namely the DJI Phantom 4 and DJI Phantom 3 Professional by SZ DJI Technology Co., Ltd., Shenzhen, China, were utilized. These UAVs are equipped with high-resolution RGB cameras, enabling them to capture detailed photos in field campaigns. The campaigns took place in Heilongjiang province, Jilin province, and Liaoning province, encompassing the major types and extent of corn residue in Northeast China. A total of 1200 UAV photos were taken across these three field campaigns, serving as the training and testing data for the CRC estimation model in this study. To ensure consistent spatial alignment with the satellite images, the UAVs maintained a flying height of 50 meters during image collection. And the CRC values for UAV images are calculated using OSTU^30^ algorithm which could distinguish the corn residue and non-corn residue easily. And the optimal threshold value is determined adaptively by the maximum variance between corn residue pixels and non-corn residue pixels within UAV image using OSTU segmentation method. Finally, the ratio between the number of segmented corn residue covered pixels and the total number of pixels in the given photo is done for CRC calculation.

#### Collecting from google earth images

For filling up the samples to full strength in 2013, 2014, 2016–2018 when there is no sample collected from field campaigns or UAV images, there are additional samples collected from the very high spatial resolution Google Earth imagery by semi-automated visual interpretation. Moreover, the enough samples are required for training and testing machine learning model for achieving accurate CRC predictions^31^. The random stratified sampling method is used to collect CRC samples based on high spatial resolution Google Earth imagery referenced from maximum NDTI composite of Landsat images. The OSTU algorithm is used to classify the corn residue pixels from non-residue pixels considering the corn residue is usually brighter than neighboring vegetation and soil. However, there is less color difference between the corn residue and the non-residue, the collect earth online tool is used to calculate CRC value in each sampling quadrat, which is a free open source software for monitoring land cover type and land cover change developed by the Food and Agriculture Organization of the United Nations (FAO)^32^. It is common to use high-resolution imagery to validate fraction maps from various ecosystems, including woody and canopy cover^33,34^. There are 49 plots of 2 m × 2 m distributed equally within each 90 m × 90 m square, and all plots are used to observe presence/absence of corn residue by visual interpretation. In each quadrat, three independent experts with local experience estimate the CRC by visual interpretation from Google Earth images. And the mean value of the three calculated CRC values represents the CRC value for the specific square. The criteria for samples collection are as followed.

**Fig. 2:**
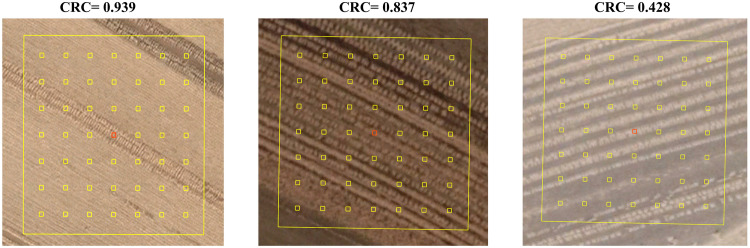
CRC sampling plots on Google Earth images.

1. The samples are selected using a random stratified sampling strategy based on the Landsat-8 maximum NDTI composite during 20th October to 10th December (after harvesting with no snows in cropland) or during 20th March to 10th May (before sowing in the next growing season) in each agricultural zone, respectively.
2. The size of samples is 0.81 ha with 90 m × 90 m, which is the size of 3 pixels in 30-m resolution Landsat images.
3. There are three independent experts with local experience calculate the CRC value using 7 × 7 plots in each quadrat with the size of 2 m × 2 m for each plot. The illustration of quadrat and plots is as Fig. 2. These 2 m × 2 m plots are used to observe presence/absence of the corn residue for calculating CRC value. And the mean value of them is used as the CRC value in given quadrat.

The amount of all these three kinds of samples in each year from 2013 to 2023 is shown in Table 1.

**Table 1:** The number of samples used for CRC estimation.

Year	2013	2014	2015	2016	2017	2018	2019	2020	2021	2023
After harvesting samples	65	140	72	88	139	62	152	112	327	—
Before sowing samples	175	76	94	91	75	172	194	310	200	641
Total samples	240	216	166	179	214	234	346	422	527	641

In the samples from 2013 to 2021, 70% were used for training the model, and 30% were used for validation. The data from the actual test samples in 2023 will be used for independently verifying the reliability of the model.

#### Remote sensing images and DEM data

There are two kinds of remote sensing images used for CRC estimation including Landsat-5/7/8/9 (https://www.earthdata.nasa.gov/) and MODIS (https://www.earthdata.nasa.gov/). The bands of blue, green, red, NIR, SWIR1 and SWIR2 of Landsat-5/7/8/9 and MODIS images are combined for synthesizing Landsat-like reflectance dataset for CRC estimation in regional Northeast China. And the details of image synthesizing using the HISTARFM algorithm is as shown in Section HISTARFM algorithm for synthesizing Landsat and MODIS images.

Considering the effect of topography on crop planting and management, DEM data is also used for CRC estimating modelling. The DEM data comes from the global 30 m resolution DEM data, released by NASA (https://www.earthdata.nasa.gov/), and the data can be used on GEE by “USGS/SRTMGL1_003”. The topographic indices including slope, aspect, elevation, and Topographic Wetness Index (TWI)^35^ are calculated based on DEM data pixel by pixel.

### Estimation of CRC

**Fig. 3:**
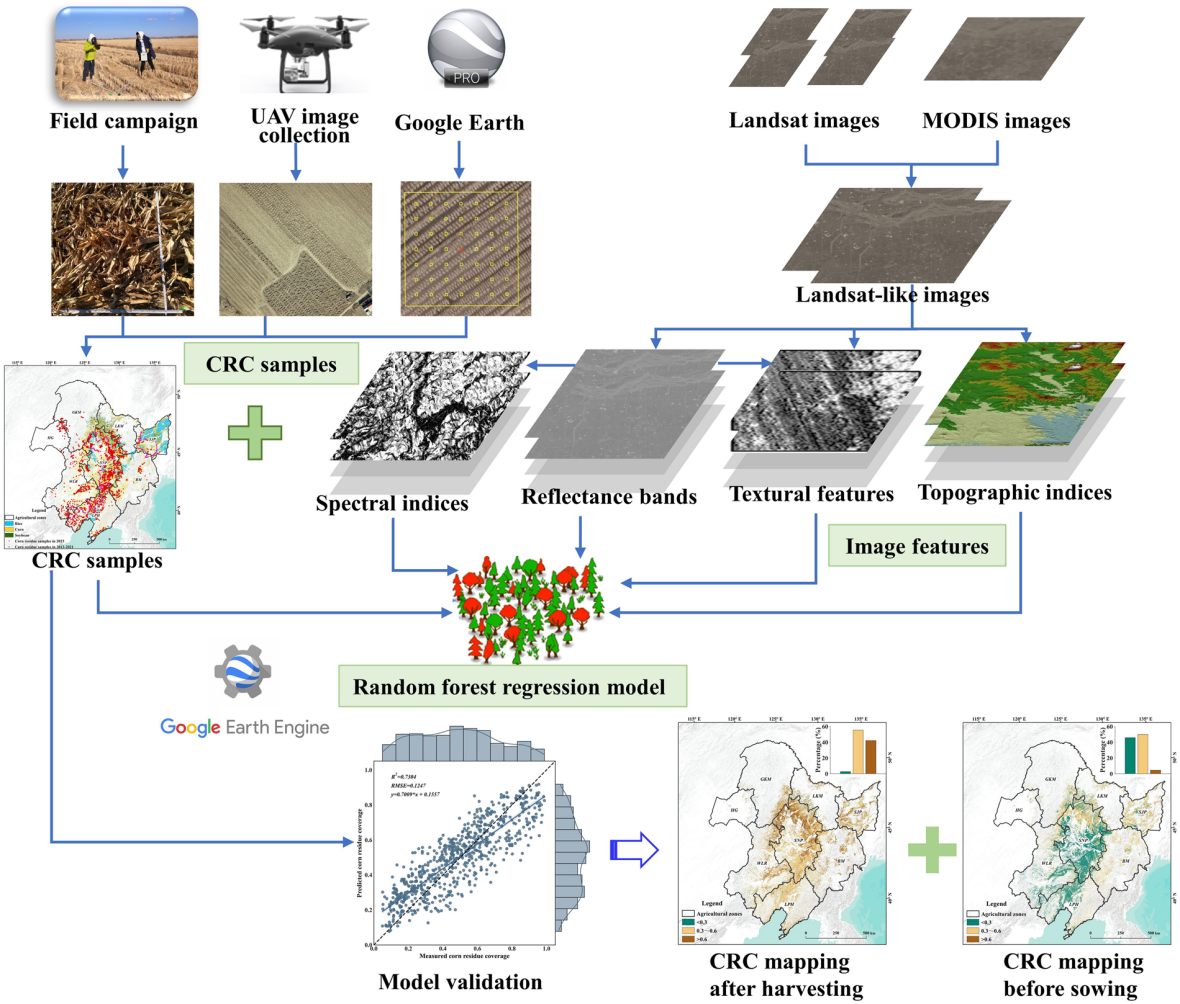
Flowchart showing the production and validation of CRC dataset in Northeast China.

The workflow of CRC estimation from 2013 to 2023 in Northeast China is presented schematically in Fig. 3. And the significant steps to produce the CRC dataset are described as follows.

1. Samples calculation of CRC from photos taken in field campaign, UAV images and high spatial resolution images from Google Earth.
2. Synthetization of Landsat and MODIS images using HISTARFM algorithm for producing time-spatial-continuous images from 2013 to 2023 in Northeast China.
3. Random forest modelling for CRC estimation and validation. There are 70% of all samples used for training random forest model resulting from image features and calculated CRC value from samples, and there are 30% of them used for validation of CRC estimation.
4. The data were validated independently using the samples collected in April of 2023, and our products were validated using statistical yearbooks.

#### HISTARFM algorithm for synthesizing Landsat and MODIS images

Due to the contamination of cloud and snow and the limitation of satellites’ revisiting period, Landsat-5/7/8/9 images cannot cover the whole study area fully in a short time with no CRC change. And the MODIS reflectance images are used to produce Landsat-like images by bring time trajectory for given image pairs. The HISTARFM algorithm derived from Kalman filter and Bayesian estimation can be used to combine two estimators synergistically to fill the gap and reduce the bias of spectral reflectance pairs^36^. And this algorithm has the applicability for gap-filling and fusing the land surface reflectance at a continental scale, which can generate gap-free monthly reflectance products at 30 m resolution for six Landsat spectral bands. The first estimator, an optimal interpolator, produces estimated Landsat reflectance values in a given month by combining previous Landsat images in the same month and the same place, pre-computed from the available Landsat images, and a fusion of Modis and Landsat reflectance obtained from the respective satellite closest to the month^37^. The second estimator is a Kalman filter to correct the reflectance bias generated by the first estimator. Therefore, the algorithm can achieve a complete coverage of the study area with images within one month in Google Earth Engine. The synthesized continuous Landsat-like images on November (after harvesting) and April (before sowing) of each year are used to estimate CRC from 2013 to 2021 in Northeast China. The Kalman filter and the Bayesian estimation could correct the bias of the first estimation.

1 $${K}_{k}={P}_{k}^{-}{H}^{T}{\left(H{P}_{k}^{-}{H}^{T}+R\right)}^{-1}$$

2 $${X}_{k}={X}_{k}^{-}+{K}_{k}\left({Z}_{k}-H{X}_{k}^{-}\right)$$

3 $${P}_{k}=\left({\rm{I}}-{K}_{k}{\rm{H}}\right){P}_{k}^{-}$$

where, ***k*** denotes dynamic variables at the ***k*** month, ***X***^***−***^ is the priori estimate, ***K*** is the Kalman gain, ***P***^**−**^ is the error covariance of the prior estimate, ***H*** is the observation operator that describes how model outputs relate to observation, ***R*** is the Landsat error covariance, ***X*** is the corrected reflectance, and ***P*** is the error covariance of the posterior estimate, ***Z*** is the observation value.

To generate the ${{\boldsymbol{X}}}_{{\boldsymbol{k}}}^{-}$, Landsat images and MODIS images were used based on Bayesian Model Averaging (BMA) approach. Firstly, the least squares solution is used to bridge the gap between Landsat and disaggregation Modis images for the selected year. Secondly, combining Modis and Landsat climatology (mean and variance of the 10 years preceding month *k*) images, using the BMA model, the ${{\boldsymbol{X}}}_{{\boldsymbol{k}}}^{-}$ can be computed.

4 $${X}_{k}^{-}=\overline{{Z}_{k}}\frac{{{\rm{P}}}_{k,MOD}}{{\bar{P}}_{k,LS}+{P}_{k,MOD}}+{u}_{k,MOD}^{30}\frac{{\bar{P}}_{k,LS}}{{\bar{P}}_{k,LS}+{P}_{k,MOD}}$$

5 $${P}_{k}^{-}=\left(1-\Upsilon \right){\left(\frac{1}{{\bar{P}}_{k,LS}}+\frac{1}{{P}_{k,MOD}}\right)}^{-1}$$

$\overline{{{\boldsymbol{Z}}}_{{\boldsymbol{k}}}}$ is the Landsat climatological mean of the 10 years prior to month ***k***, ${\bar{{\boldsymbol{P}}}}_{{\boldsymbol{k}},{\boldsymbol{LS}}}$ is the Landsat climatological variance of the 10 years prior to month ***k***, ***ϒ*** is a fraction of the error covariance of the estimate that is attributed to bias, and ***P***_***k,MOD***_ is the variance of the downscaled Modis reflectance. Thirdly, according to the previous study^38^, ***ϒ*** is determined empirically and the value has been reported lower than 1. In this section, we set ***ϒ*** = 0.6, which cloud captures the trade-off between land cover pixels that change rapidly and tend to have highly biased reflectance (cropland), and pixels that change slowly (unmanaged forest)^36^. Finally, we can get the gap-free monthly images which has high correlation with monthly Landsat composite and capture the pixels feature.

#### Combination of image features

Considering the spectral and textural difference between corn residue and non-residue, the combination of spectral indices, reflectance bands, and texture features is used for CRC estimation. The popular NDTI, NDI5, NDI7, NDSVI, STI, SGNDI, DFI, BI3, MCRC, and NDRI are used in spectral indices group. And the GLCM features are calculated consists of 18 bands per input band if directional averaging is on and 18 bands per directional in the kernal. And Angular Second Moment, Contrast, Correlation, Variance, Inverse Difference Moment, Sum Average, Sum Variance and Sum Entropy will be calculated if not. The effect of topography on crop planting and management is considered using Elevation, Slope, Aspect and TWI. All features are shown in Table 2. Feature selection is a crucial aspect in the estimation of CRC as it significantly impacts the efficiency and effectiveness of the CRC estimation. Recursive Feature Elimination (RFE) is a feature selection algorithm, which begins by searching for a subset of features from the complete set available in the training dataset. It then proceeds to eliminate features until the desired number is retained iteratively. The goal is to expedite model training and improve its generalization ability. Hence, the RFE method is used to do feature selection in this study.

**Table 2:** Combination of image features used for CRC estimation.

	Features			
	Name	Abbreviation	Formula	References
**Spectral indices**	Normalized difference tillage index	NDTI	(SWIR1−SWIR2)/(SWIR1 + SWIR2)	^15^
	Normalized difference index 5	NDI5	(NIR−SWIR1)/(NIR + SWIR1)	^48^
	Normalized difference index 7	NDI7	(NIR−SWIR2)/(NIR + SWIR2)	^48^
	Normalized difference senescent vegetation index	NDSVI	(SWIR1−Red)/(SWIR1 + Red)	^13^
	Simple tillage index	STI	SWIR1/ SWIR2	^15^
	Shortwave Green Normalized Difference Index	SGNDI	(Green −SWIR2)/(Green + SWIR2)	^16^
	Dead fuel index	DFI	100 × (1- SWIR2/ SWIR1) × (Red/NIR)	^49^
	Three-band index	BI3	(SWIR2- Red) × (SWIR2 + SWIR1)	^24^
	Modified crop residue cover	MCRC	(SWIR1−Green)/(SWIR1 + Green)	^50^
	Normalized difference residue index	NDRI	(Red−SWIR2)/ (Red + SWIR2)	^14^
**Reflectance bands**	**Name**		**Number**	
	Blue Green Red NIR SWIR1 SWIR2		6	
Textural features	GLCM (6 reflectance bands) 6 × 18		108	
Topographic indices	Elevation, Slope, Aspect, TWI		5	

#### Random forest regression modelling

RF is an ensemble-learning algorithm^39^ that has been widely used to estimate CRC^40,41^ and aboveground biomass (AGB)^42,43^ due to its excellent performance. By using the grid searching method, the optimal parameters of the random forest regression are determined by considering the differences in the number of samples in each agricultural zones. The samples within each agricultural zone are divided into training dataset (70%) and a test dataset (30%) by stratified spatial random sampling, and the model accuracy evaluation is achieved by 10-fold cross-validation. Additionally, it calculates a relative importance score for each predictor variable, which reflects its contribution to the RF model. Moreover, the measured samples in April 2023 are used for validation of accuracy and robust. And the 30 m crop classification results of Northeast China in our previous study are used to mask corn cropland. Considering there is no crop classification results within WLR, HG, and part of GKM in the previous study, we use the same method to map the corn cropland in these areas.

At the same time, using actual field-measured CRC data in Northeast China in 2023 for independent verification, test the robustness of the model over time, and use statistical indicators R^2^ and RMSE to evaluate the model performance.

6 $${R}^{2}=1-\frac{{\sum }_{i=1}^{n}{({y}_{i}-{\widehat{y}}_{i})}^{2}}{{\sum }_{i=1}^{n}{({y}_{i}-{\bar{y}}_{i})}^{2}}$$

7 $$RMSE=\sqrt{\frac{1}{n}{\sum }_{i=1}^{n}{\left({y}_{i}-{\widehat{y}}_{i}\right)}^{2}}$$

In the formula, *y*_*i*_ is the measured CRC. And ${\widehat{y}}_{i}$ is the estimated CRC using a multiple linear regression model and random forest model.

## Data Records

The annual CRC mapping after harvesting and before sowing in the next growing season are prepared using the defined dataset and can be accessed on the public repository Figshare 10.6084/m9.figshare.23993517.v4^44^. The data can be imported into remote sensing processing software (e.g., ENVI), and standard geographical information system software (e.g., ArcGIS). The validation data consisted of two parts: (i) CRC data calculated by Li *et al*. and (ii) Conservation Tillage Statistics Yearbook.

## Technical Validation

### Validation of synthesized Landsat-like images

**Fig. 4:**
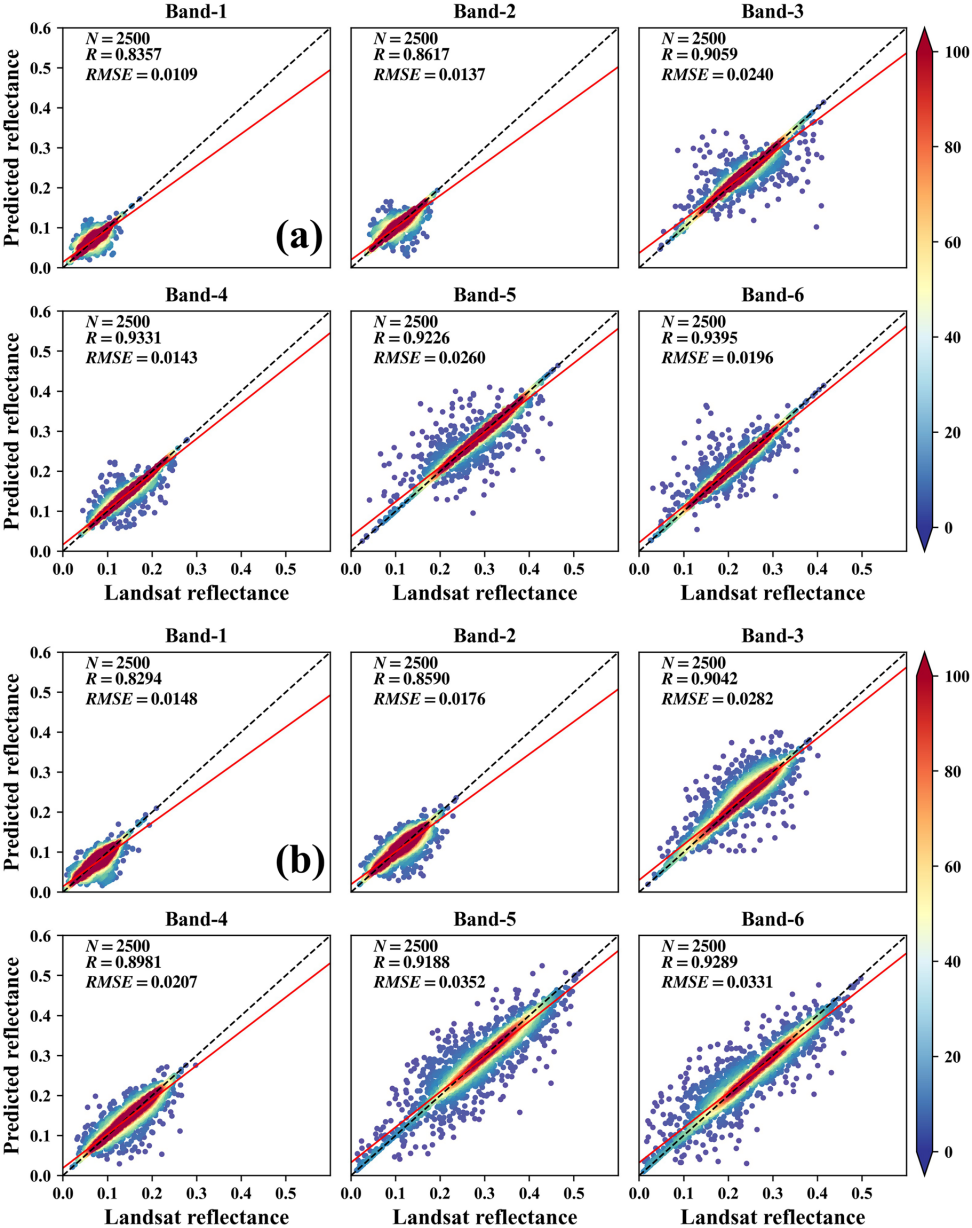
Comparation of synthesized reflectance after harvesting (**a**) and before sowing (**b**).

To evaluate the synthesized reflectance using the HISTARFM algorithm, we compare the image reflectance before and after synthesisation. There are 2500 cropland samples from synthesized images after harvesting and before sowing are used for validating the synthesized reflectance. Figure 4 is the comparation of synthesized reflectance after harvesting (a) and before sowing (b), which shows that all scatter plots are concentrating on the 1:1 line. Comparably speaking, the synthesized accuracy of Band-1 and Band-2 are lower than that of Band-3 to Band-6. And the R values of synthesized reflectance of Band-3 to Band-6 are all greater than 0.90. All these results in Fig. 4 reveal that there are high correlations between Landsat reflectance with the synthesized Landsat-like reflectance.

### Accuracy assessment of CRC estimation

**Fig. 5:**
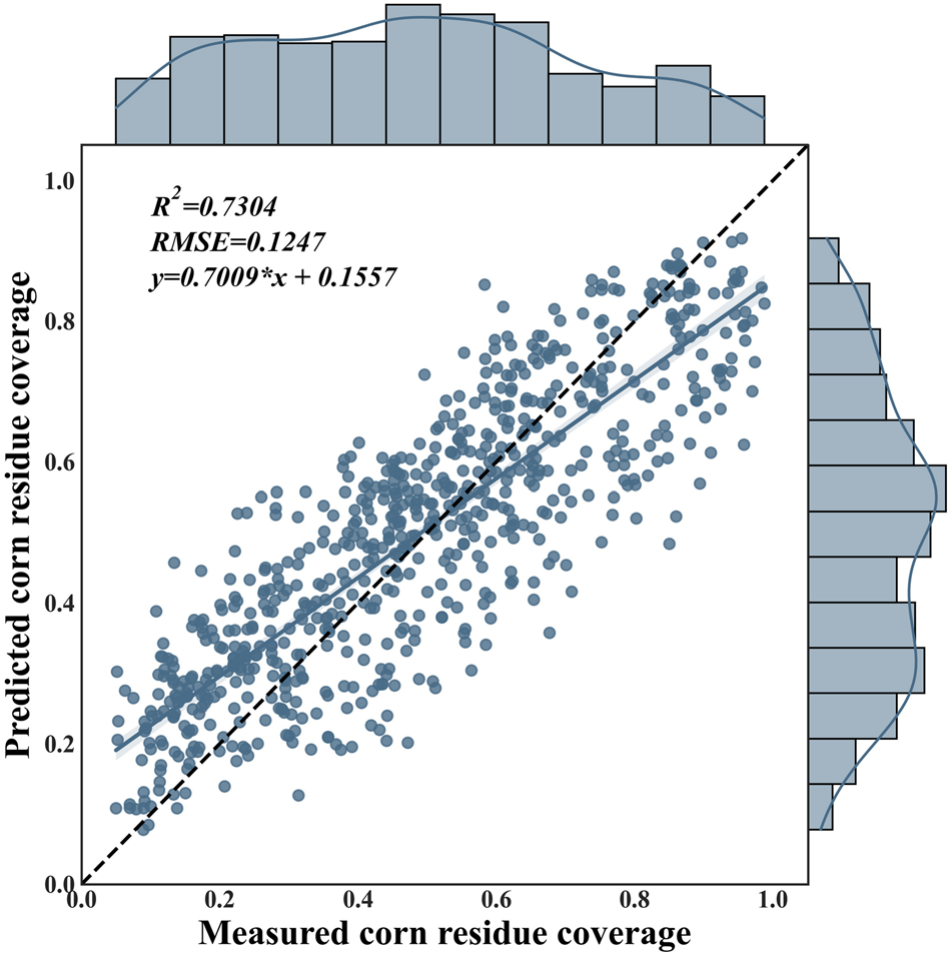
Accuracy assessment of CRC estimation using testing samples. Notes: The marginal kernel density plots above and to the right of the scatterplot show the distribution of the data in one dimension.

Figure 5 shows the accuracy assessment result of CRC estimation using testing samples. We can conclude that our CRC estimation model displays a good performance in CRC estimation in Northeast China. Firstly, the correlation between measured and predicted CRC is high with R^2^ of 0.7304 and RMSE of 0.1247. Secondly, the scatter points are concentrated on 1:1 line with a wide dynamic range from 0.0 to 1.0. In addition, we analyzed the importance of all characteristics of the models. The analyzed results of characteristic importance show that the largest contributions in the model are NDTI and STI, which is consistent with the results of previous studies^23,26^.

**Fig. 6:**
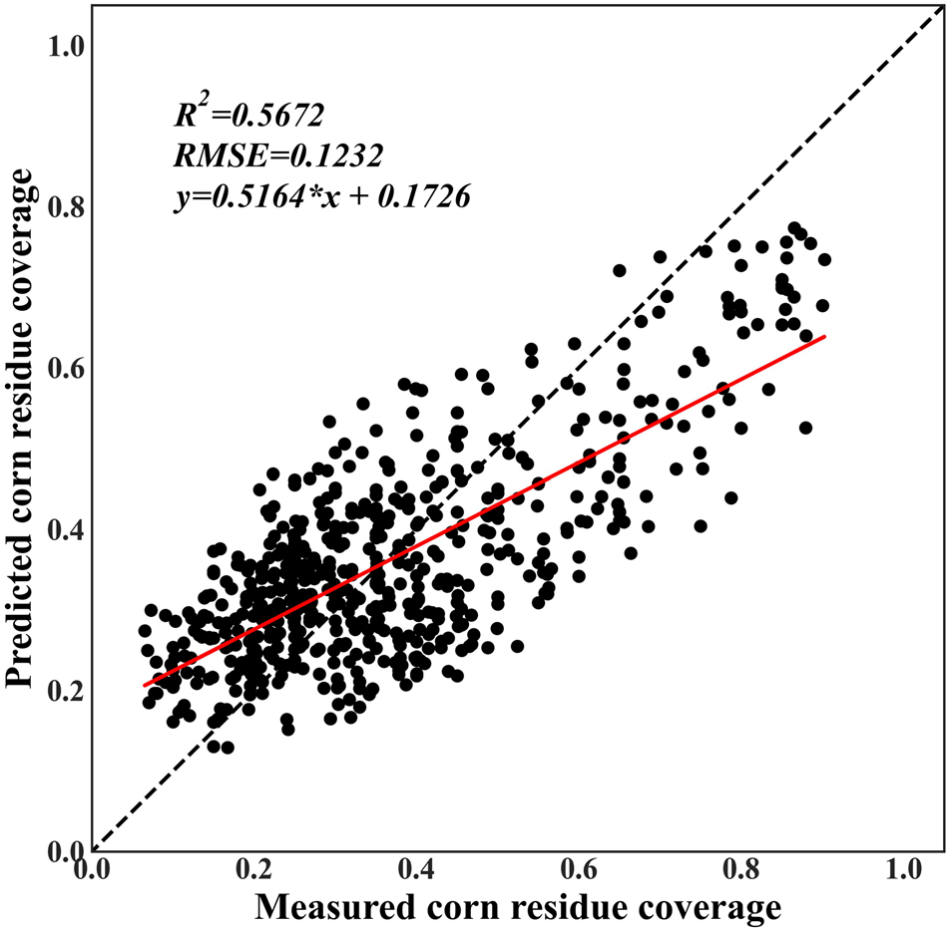
Accuracy assessment of CRC estimation using independent measured CRC data in 2023.

Furthermore, the measured CRC data in 2023 which is not used for training model is used to validate the accuracy the CRC estimation during 2013–2021. Figure 6 is the accuracy assessment of CRC estimation using independent measured CRC data in 2023. And the R^2^ of measured CRC and estimated residue coverage in 2023 before sowing is 0.5672, which reveals the good performance of CRC estimation in this study. The independent validation result using the samples measured in 2023 which is not used for model training reveals that the predicted CRC is correlated with the measured CRC with R^2^ is 0.5672. Comparably speaking, the predicted CRC is a little lower than the measured CRC, which is due to the measured CRC is collected at the end of March and the beginning of April in 2023, while the modelling is done using the samples collected in the mid to late April from 2013 to 2021.

### Wall-to-wall comparison with published results

**Fig. 7:**
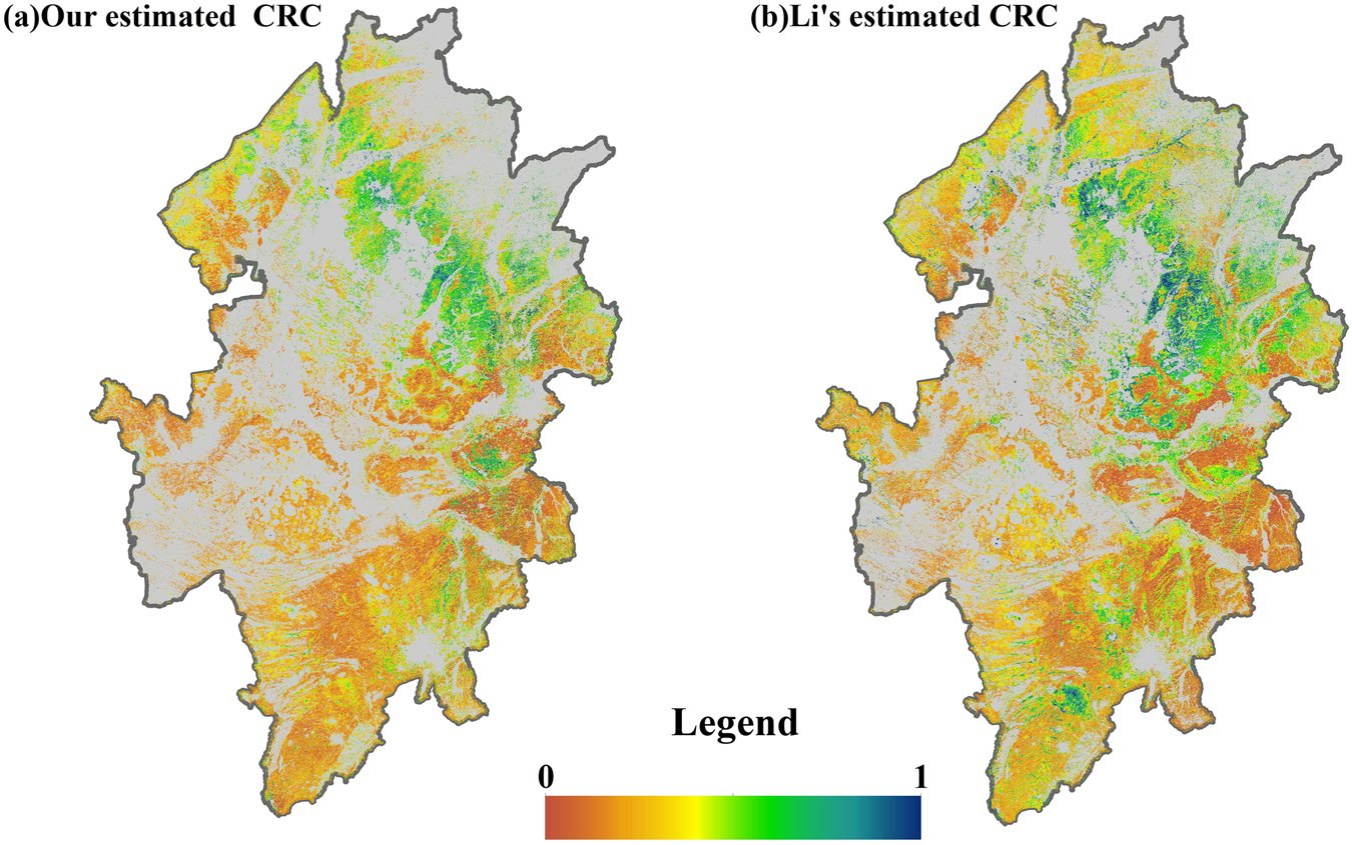
Comparison with the published estimated CRC of Li *et al*. in Songnen Plain.

For validating the CRC estimation results further, the wall-to-wall comparison in Songnen Plain is done with the published results of CRC estimation from Li *et al*.^28^. Li *et al*. estimate the CRC using Sentinel-2A images in Songnen Plain in 2019–2022. The spatial resolution of Li’s CRC estimation result is 20 m, and the spatial resolution of our CRC estimation using Landsat 5/7/8/9 is 30 m. So, the nearest neighbour method is done to resample the CRC estimation of Li *et al*. to 30 m. Figure 7 is the spatial trend of comparison with published results in Songnen Plain of Li, which reveal similar spatial trends between the CRC estimation results of Li *et al*. with our estimation results in this study.

**Fig. 8:**
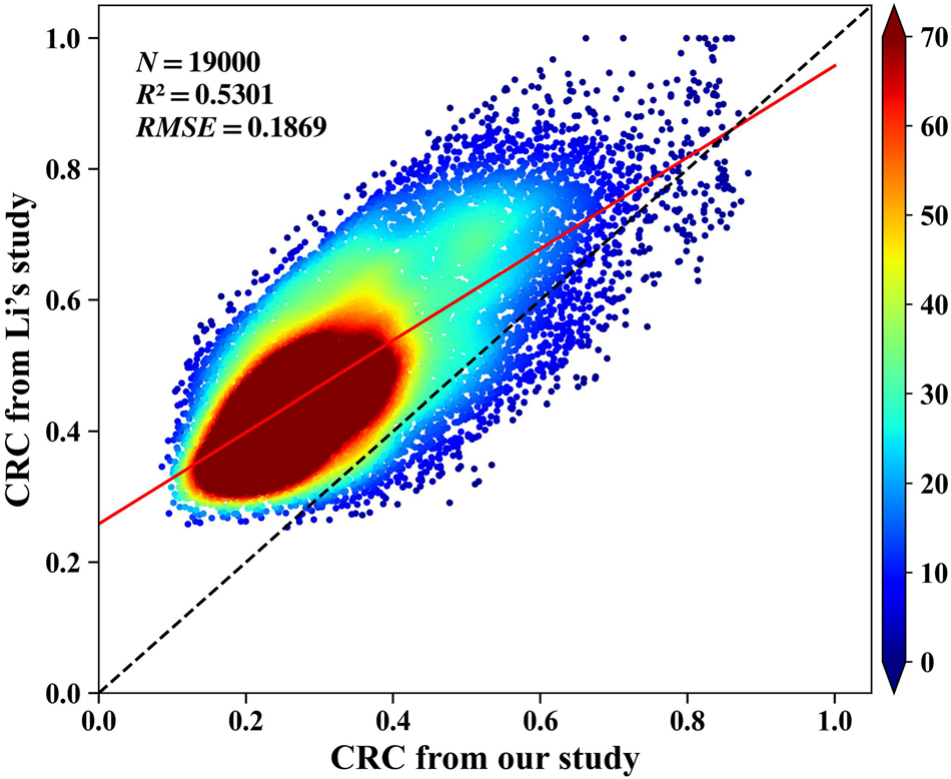
Comparison with published estimated CRC of Li *et al*. in Songnen Plain using correlation analysis.

Figure 8 is the quantitative comparation of CRC estimation results between Li *et al*. and our results in this study. The R^2^ of Li’s CRC estimation is 0.7292 using the best performing model with the optimized spectral index in Songnen Plain. And we estimate CRC with the R^2^ of 0.7304 in the whole of Northeast China. In addition, we selected 19000 samples randomly to validate the correlation CRC estimation results between Li’s study. and our results in this study. Figure 8 reveals that the correlation coefficient R is 0.7281 and RMSE is 0.1869. From Fig. 8, it can be seen that the estimated CRC of Li *et al*. is significantly higher than our estimated result, and the difference might be due to the time difference and the modelling difference. Firstly, the samples of Li *et al*. were acquired at the end of March, and the samples acquired were higher coverage samples, while the time of acquiring the samples for this study is basically in the middle to late April to the beginning of May, and the CRC is less than that in March, which makes the final straw coverage of our products lower than the previous study. The measured CRC sample is an important variable that affects the accuracy of the model for estimating CRC^43,45^. Secondly, the features used for modelling and trained model were different, too. Li *et al*. used NDTI only for CRC estimation using a linear regression. In this study, we used 10 tillage indices, 6 spectral bands, 108 texture features, and 5 terrain features with random forest regression to build the CRC estimation model. Wang *et al*.^41^ found that the accuracy of CRC estimation is higher than that of linear models. Furthermore, our published study results also revealed that the accuracy of CRC estimation using random forest was higher than that using linear regression method in estimating the CRC in Northeast China^26^.

### Comparison with official statistical data of conservative tillage

**Fig. 9:**
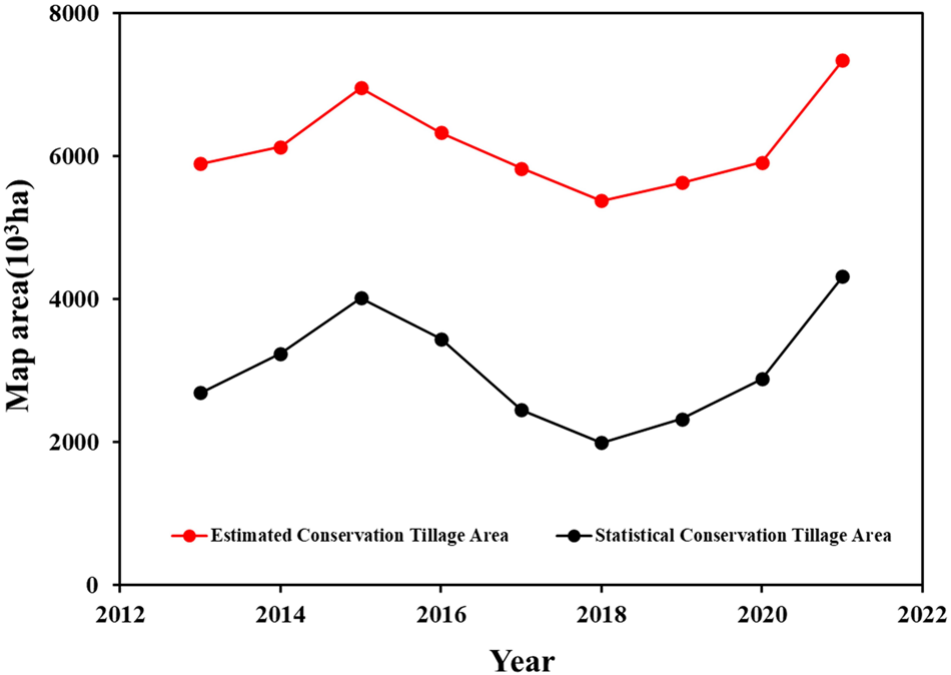
Changing trends of conservation tillage area and CRC value higher than 0.3 in Northeast China.

Since there is no official statistical data on CRC in Northeast China, we compare and validate our CRC results with official statistical data of conservation tillage area in *China Agricultural Machinery Industry Yearbook* and *China Agricultural Mechanization Yearbook*. Figure 9 shows the changing trends of conservation tillage area and CRC value higher than 0.3(Defined as conservation tillage) in Northeast China. And Fig. 9 reveals that there are similar changing trajectories for conservation tillage area and CRC value higher than 0.3 during 2013–2021. All of them are increasing from 2013 to 2015 and there is a local peak in 2015. There is an official policy of crop planting strategy adjustment in 2016, and some corn planted area is changed into soybean or rice paddy. Therefore, there is a decrease for conservation tillage area and CRC value higher than 0.3 in 2017 and 2018. And the conservation tillage area and CRC value higher than 0.3 is increasing again from 2018. The reasoning for this increasing is that the Ministry of Agriculture and Rural Affairs issued the policy of *Outline for the Protection of Black Soil in Northeast China (2017*–*2030)* (http://www.moa.gov.cn/nybgb/2017/dqq/201801/t20180103_6133926.htm) in 2017. And the conservation tillage techniques were encouraged by financial subsidies. Furthermore, the increasing rate of conservation tillage area has accelerated from 2020. The reasoning for this accelerating is the implementation of *Action Plan for the Protection of Black Soil in Northeast China (2020*–*2025)* (http://www.moa.gov.cn/gk/tzgg_1/tz/202003/t20200318_6339304.htm), which promote the application of conservation tillage in Northeast China.

**Fig. 10:**
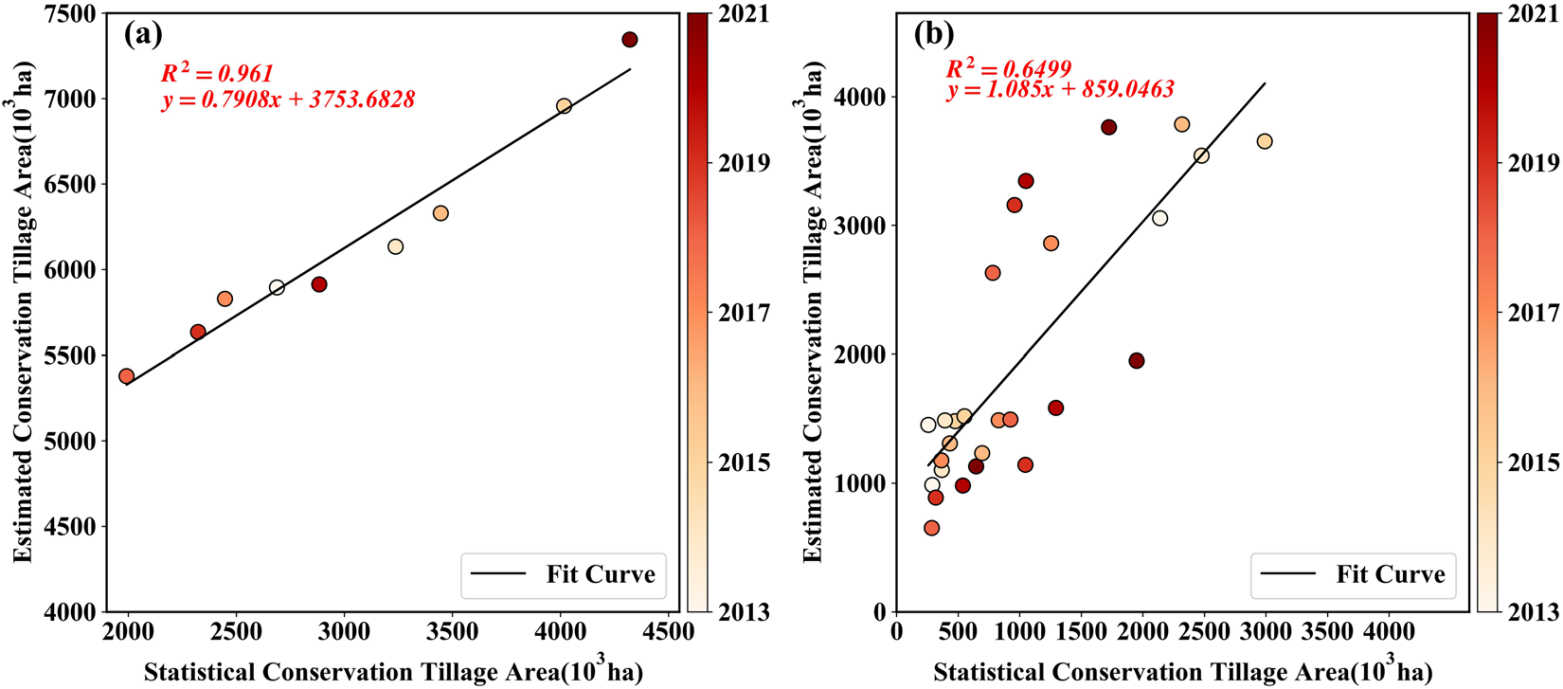
Comparison of the estimated area within CRC > 0.3 with the statistical conservative tillage area on region scale (**a**) and provincial scale (**b**) from 2013 to 2021.

Figure 10 is the comparison of the estimated area within CRC > 0.3 with the statistical conservative tillage area on region scale (a) and provincial scale (b) from 2013 to 2021. Figure 10a revealed that our estimated area with CRC greater than 0.3 was highly correlated with the statistical conservation tillage data of three provinces in Northeast China with R^2^ of 0.9610. Compared with the statistical data, the area of conservation tillage estimated in this study is higher, and the possible reasons were as follows. Firstly, only the conservation tillage area of mechanical tillage was counted in *China Agricultural Machinery Industry Yearbook*, and the conservation tillage area from the small holder cropland was not included in it^46^. Secondly, there was time difference between samples collection and statistical data. The conservation tillage area counted in the statistical yearbook was always at the beginning of May after sowing, but the CRC measurement was always at the middle of April before sowing.

### Comparison with google earth images

**Fig. 11:**
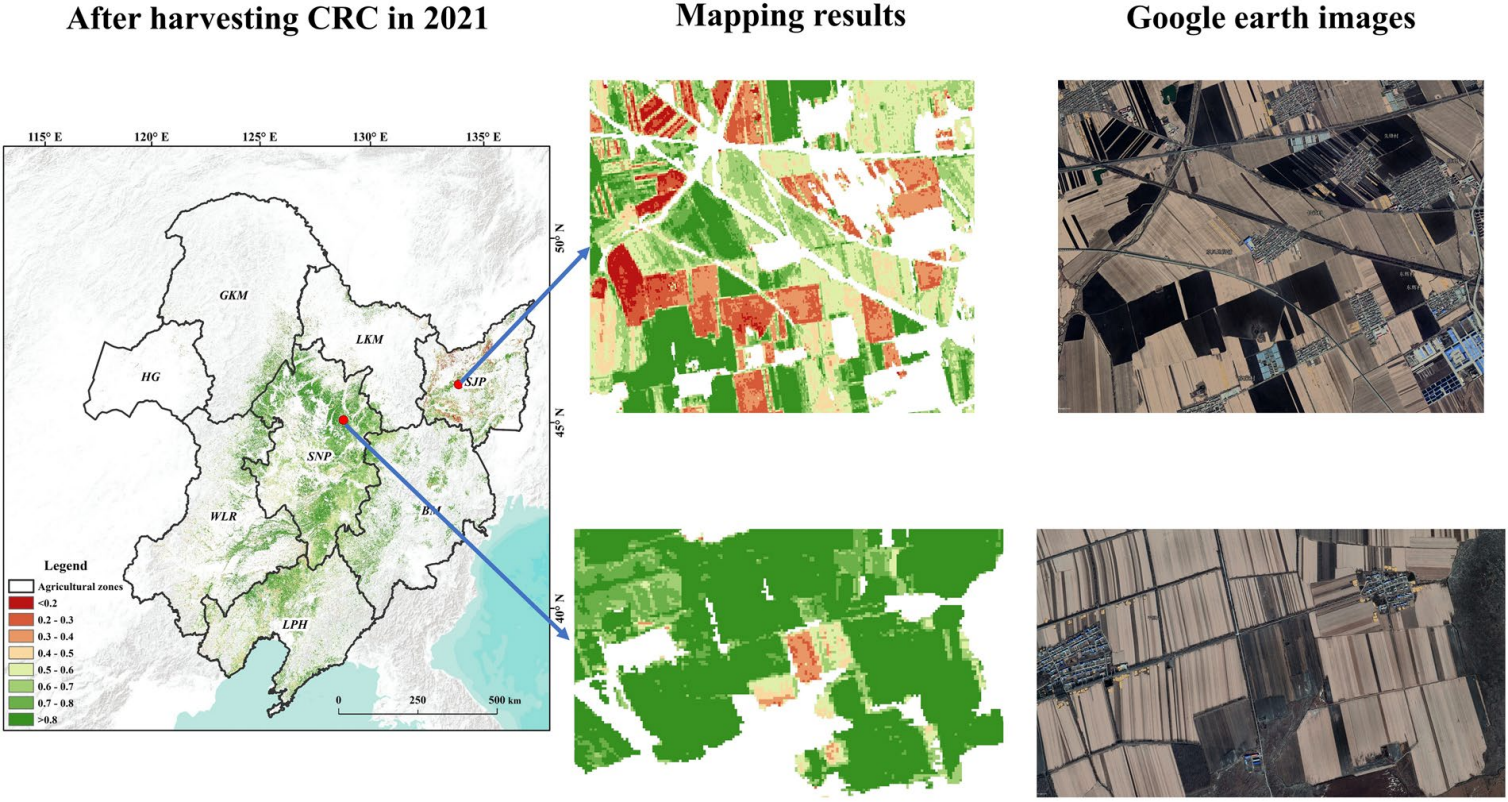
Comparison between the maps of CRC and high-resolution images from Google Earth in the study area.

To further validate the accuracy of the CRC results, we conducted a comparison with high spatial resolution images obtained from Google Earth (Fig. 11 and Table 3). The results revealed a remarkable consistency between the spatial details of our CRC outputs and the high-resolution images available on the Google Earth platform. Additionally, the comparison between the observed and estimated CRC values revealed a negligible difference, suggesting that the CRC obtained through visual interpretation and remote sensing are highly consistent. This finding underscores the reliability of the CRC data and supports their suitability for use in subsequent research endeavors.

**Table 3:** Labels resulting from visual interpretation of high spatial images from Google Earth used for CRC validation.

Location	Mapping Results	Google Earth images
124.780856°E 43.608082°N	CRC = 0.658	
125.248449°E 43.653694°N	CRC = 0.682	
125.945043°E 44.168036°N	CRC = 0.666	
124.710024°E 43.536296°N	CRC = 0.55	

### Discussion of limitation and future work

We acknowledge that there are still some shortcomings in this study. (1) A limitation to the resultant maps of CRC is from that there should be more samples for model training and testing. In general, machine learning models require large training and testing datasets to achieve accurate predictions^31^. Unfortunately, there are insufficient samples for modelling. If more samples are collected, the CRC estimated results would be more robust. (2) Due to the very short time window for CRC estimation after harvesting, the fused images from Landsat 5/7/8/9 and MODIS are used to estimate CRC, and the image fusion error will propagate to the CRC estimation. In Northeast China, the corn is harvested in middle October, and it will be snow in the middle of November. It is very difficult to collect enough Landsat 7/8 image in this short time in large regional area of Northeast China. So we fused Landsat 5/7/8/9 and MODIS images for the fully covered CRC estimated result. Although the fused images are able to predict reflectivity well, the fused images are still blurry due to the difference in spatial resolution between Landsat and MODIS, which makes it difficult for the fusion algorithm to capture texture-rich features^47^.

## Usage Notes

### Spatiotemporal distributions of CRC

**Fig. 12:**
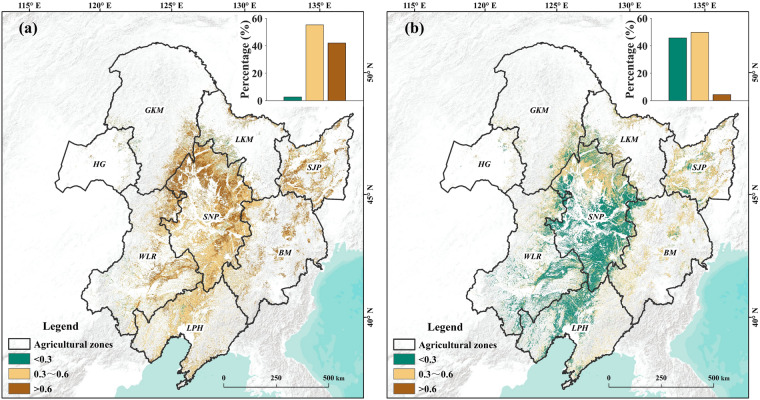
Spatial distribution of average CRC after harvesting (**a**) and before sowing (**b**) in Northeast China from 2013 to 2021.

**Fig. 13:**
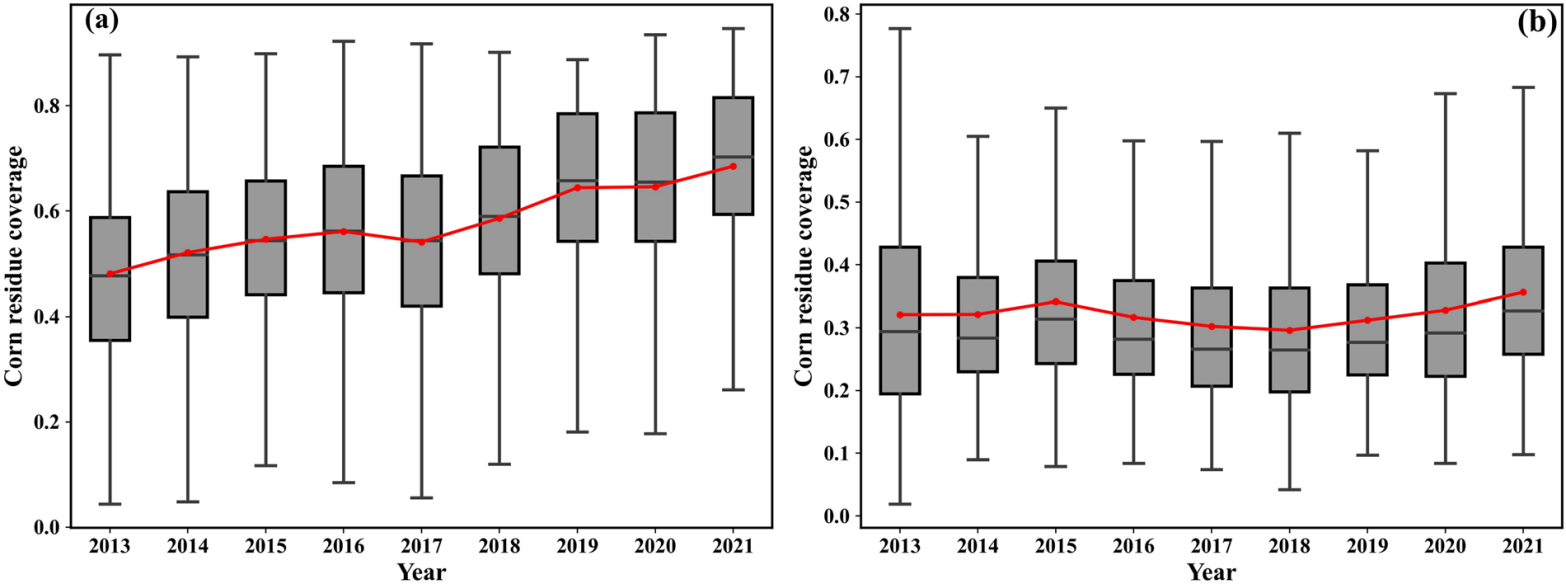
Temporal changing of CRC values after harvesting (**a**) and before sowing (**b**).

The spatial distribution of averaged CRC after harvesting and before sowing within 2013–2021 in Northeast China is as Fig. 12. And Fig. 12 reveals that the CRC after harvesting than that before sowing. The most area is covered with CRC value within 0.3–0.6 and only very few areas are covered with CRC value less than 0.3 after harvesting. However, there are more areas are covered with CRC value less than 0.3 and the proportion of area with CRC value within 0.3–0.6 and greater than 0.6 is decreased before sowing in the next growing season. This decreasing maybe resulted from the phenomenon of corn residue decomposition, removing by farmer for sowing. The high CRC areas are mainly concentrated in Songnen Plain after harvesting. Moreover, the CRC value in south of study area is lower than that in north area because the sowing date in south area is earlier than that in north area and there are more corn residues are removed in south area. The temporal changing of CRC values after harvesting and before sowing is as Fig. 13. We can conclude that the CRC is increasing from 2013 to 2021 from Fig. 13.

## Data Availability

The programs used to generate all the results were Python (3.10) JavaScript and ArcGIS (10.8). Analysis scripts used in this study will be available at 10.6084/m9.figshare.23993517.v4^44^.
